# The effects of virtual reality game training on trunk to pelvis coupling in a child with cerebral palsy

**DOI:** 10.1186/1743-0003-10-15

**Published:** 2013-02-07

**Authors:** Gabor J Barton, Malcolm B Hawken, Richard J Foster, Gill Holmes, Penny B Butler

**Affiliations:** 1 Research Institute for Sport and Exercise Sciences, Liverpool John Moores University, Byrom Street, Liverpool L3 3AF, UK; 2 North West Movement Analysis Centre, Alder Hey Children’s NHS Foundation Trust, Eaton Road, Liverpool L12 2AP, UK; 3 The Movement Centre, Robert Jones and Agnes Hunt Hospital, Oswestry, SY10 7AG, UK

**Keywords:** Virtual rehabilitation, Cerebral palsy, Core control, Pelvis and trunk coupling

## Abstract

**Background:**

Good control of trunk and pelvic movements is necessary for well controlled leg movements required to perform activities of daily living. The nature of movement coupling between the trunk and pelvis varies and depends on the type of activity. Children with cerebral palsy often have reduced ability to modulate coupling between the trunk and pelvis but movement patterns of the pelvis can be improved by training. The aim of this study was to examine how pelvis to trunk coupling changed while playing a computer game driven by pelvic rotations.

**Methods:**

One boy with cerebral palsy diplegia played the Goblin Post Office game on the CAREN virtual rehabilitation system for six weeks. He navigated a flying dragon in a virtual cave towards randomly appearing targets by rotating the pelvis around a vertical axis. Motion of the pelvis and trunk was captured in real-time by a Vicon 612 optoelectronic system tracking two clusters of three markers attached to the sacrum and thoracic spine.

**Results:**

Convex hull areas calculated from angle-angle plots of pelvic and trunk rotations showed that coupling increased over game training (*F*_*1,11*_ *= 7.482, p = 0.019*). Reaching to targets far from the midline required tighter coupling than reaching near targets (*F*_*1,12*_ *= 10.619, p = 0.007)*.

**Conclusions:**

Increasing coupling appears to be an initial compensation mechanism using the better controlled trunk to drive rotation of the pelvis. Co-contractions causing increased coupling are expected to reduce over longer exposure to training. The control scheme of the training game can be set to facilitate de-coupling of pelvic movements from the trunk. Using large ranges of pelvic rotation required more coupling suggesting that training of selective pelvic movements is likely to be more effective close to a neutral pelvic posture.

## Background

There is evidence to support the hypothesis that the pelvis and trunk play an active role in gait, as opposed to the traditional view [1] which regards the core of the body as a “passenger unit” carried by the lower extremities, which are termed the “locomotor unit”. Responses triggered by balance perturbations involve neck, trunk and thigh muscles which contract simultaneously, even before the activation of muscles around the ankle joint in unimpaired subjects [2-5]. In prospective studies [6,7], reduced strength and impaired proprioception around the core (pelvis and trunk) were found to be associated with increased risk of injuries, specifically around the knee. This suggests that good control of the movement of the core is a prerequisite for well controlled use of the legs.

Good control of the interaction between the trunk and pelvis is required to carry out activities of daily living. During level walking the nature of this interaction depends on walking speed [8-12]. At slow speeds the pelvis and trunk are coupled in the transverse plane and move in-phase, but as walking speed increases there is a transition from in-phase to anti-phase coupling [9,10]. Retraction of the trunk, together with protraction of the pelvis on the swing leg side, increases step length and therefore improves the efficiency of gait.

One of the primary problems in cerebral palsy is reduced selective control of movement [13]. Altered selective motor control of the pelvis and trunk hinders efficient gait and thereby activities of daily living. Improved ability to reduce in-phase coupling between the trunk and pelvis is expected to improve gait at normal walking speeds characterised by anti-phase coupling. Activities of daily living are also expected to benefit from the reduced in-phase coupling.

Due to their central location in the body’s linked chain of segments the trunk and pelvis are mechanically restricted to a greater degree than the extremities and the head. In spite of this constraint, movement of the trunk and pelvis can be altered through training [14,15]. There are numerous reports of successfully using custom made computer games to both test and train movement control of the upper or lower extremities [16-19]. Applications of serious gaming focused on the pelvis and trunk, however, are scarce. Our Goblin Post Office (GPO) training game [20-25] is derived from the conceptual framework of Targeted Training [26] in that it allows sequential training of body segments from upper to lower, thereby increasing successively the complexity of the motor task. We report here some findings (from a larger training study) in which we observed changes in coupling between the pelvis and trunk when the game was controlled by movement of the pelvis.

## Methods

One boy with cerebral palsy spastic diplegia (age: 10 years; height: 1.34 m; mass: 36 kg) trained for 6 weeks, twice a week for 30 minutes (13 sessions in total) on our custom-made GPO computer game, developed in the CAREN system (Motek Medical, Amsterdam, The Netherlands). The child had no history of surgical intervention, and was receiving no conventional physiotherapy or core specific rehabilitation which might have influenced his responses to game training. His Gross Motor Function Classification System (GMFCS [27]) score of 1 indicates that he walks indoors and outdoors without limitations and can run and jump but speed, balance and co-ordination may be reduced. He attends mainstream education and does not have learning difficulties. On physical examination he had a 10° equinus contracture in the right ankle and bilaterally tight hamstrings (popliteal angles of 60°). The hips were offset into internal rotation bilaterally together with external tibial torsion. Muscle strength was reduced in right dorsi/plantarflexors, right hip extensors and bilaterally in hip abductors and foot in/evertors. There were positive signs of spasticity bilaterally in the plantarflexors (Tardieu 5 on right and 2 on left side), hamstrings (Tardieu 2) and quadriceps (positive Duncan Ely signs). Ethical approval was obtained from the NHS National Research Ethics Service (07/H1014/83) and local ethics committees (Royal Liverpool Children’s NHS Trust and Liverpool John Moores University), confirming that the research carried out was in compliance with the Helsinki Declaration. The subject’s parent provided written consent for the study.

**Figure 1:**
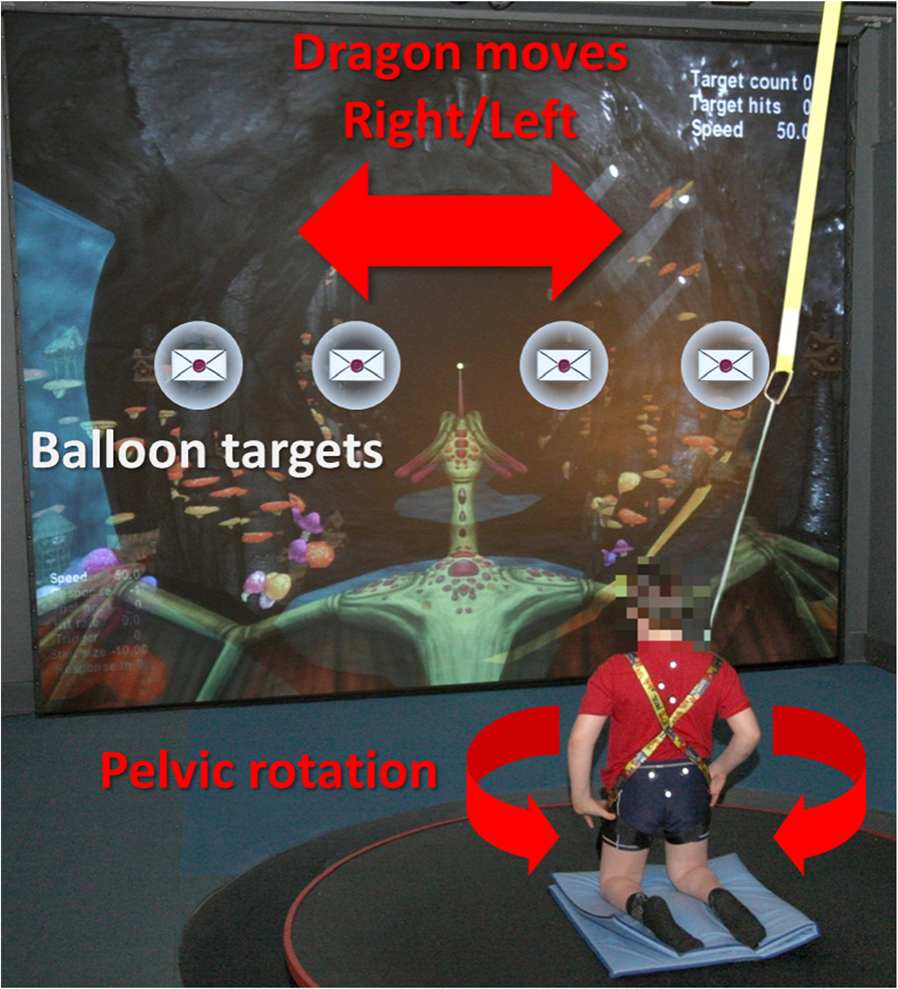
**The Goblin Post Office game. **The dragon flies forwards through the cave at a speed set by the game software, and individual targets appear at some distance ahead unpredictably in one of the four horizontal locations shown. The subject directs the dragon towards the target by left- and rightward pelvic rotation, registered by a triangular array of reflective markers. A second set of markers registers trunk motion, enabling evaluation of trunk to pelvis coupling.

The subject’s task in the game was to use the horn on a dragon’s head to burst balloons containing letters. After one balloon target was either hit or missed, the next target appeared at some distance in front of the dragon in one of four unpredictable locations (Figure 1). Forward speed of the dragon through the virtual cave was controlled by the game software, the subject controlled left- and rightward movement (speed) of the dragon by rotating the pelvis about a vertical axis in a high kneeling posture. This posture was used to simplify control requirements by eliminating the complexity of ankle motion. High kneeling ensured that the pelvis and trunk remained in the same relative posture as standing, thus giving a more realistic training opportunity. Motion of the dragon and location of the targets were confined to a single horizontal plane. Rotations of the pelvis and trunk about a vertical axis were captured by tracking the 3D orientation of two triangular clusters of three retro-reflective markers placed over the thoracic spine and on the sacrum. Neither rotation of the pelvis about the left/right axis (tilt), nor trunk rotations about either axis, had any effect on the motion of the dragon. At the beginning of the first training session the control scheme of the game was explained to the participant and he played the training level of the game which responded to rotations of the pelvis but the dragon was not moving forwards.

**Figure 2:**
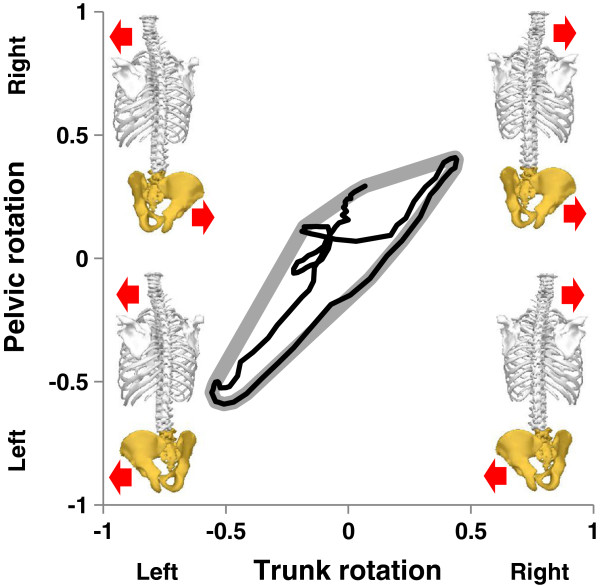
**Angle-angle plot (black line) visualising the interaction between rotation of the trunk and pelvis. **The area of the convex hull surrounding the angle-angle plot (bold grey line) was used to quantify coupling. Four quadrants of the chart represent the possible combinations of trunk and pelvic rotation.

To quantify coupling between the trunk and pelvis, angle-angle plots of trunk and pelvic rotation, normalised to their respective ranges of motion over all sessions, were generated for sections of gameplay between consecutive targets. The area of the convex hull containing all data points of the angle-angle plot was calculated using the CONVHULL function in MATLAB (The Mathworks, Natick MA, USA). This area was used to quantify the level of coupling between the trunk and pelvis (Figure 2). Low values of Area indicate tight in-phase or anti-phase coupling and high values indicate a lack of association between rotation of the trunk and of the pelvis.

## Results

**Figure 3:**
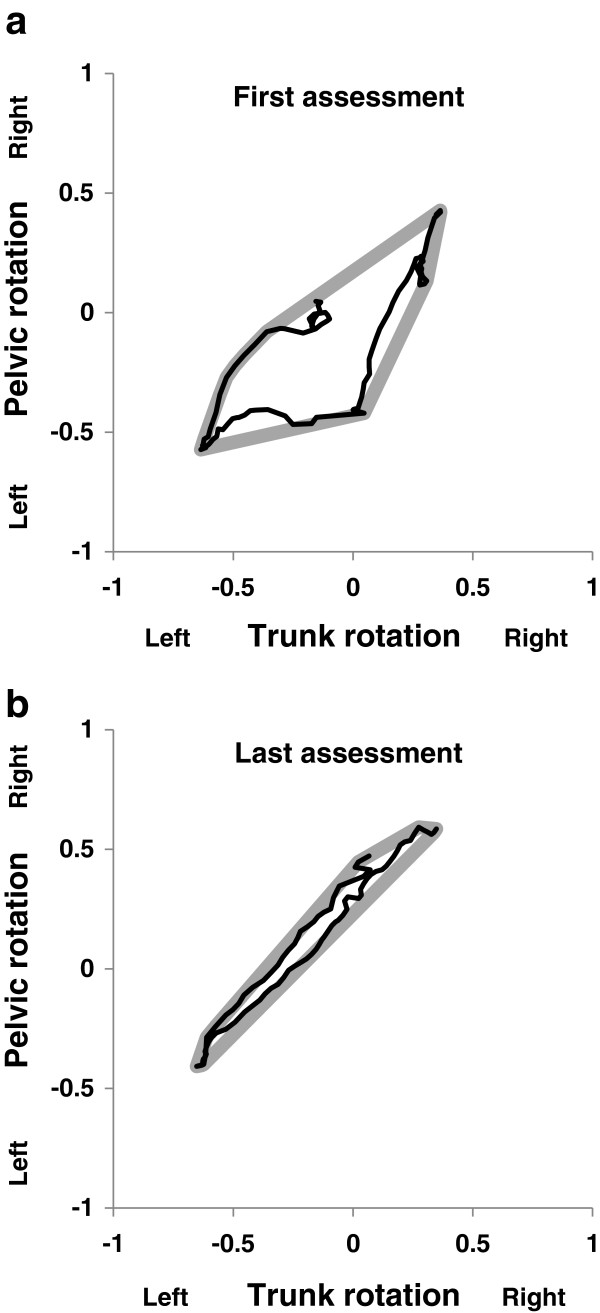
Examples of angle-angle plots (black line) and convex hulls (bold grey line) representing coupling between the trunk and pelvis a) in the first assessment and b) in the last (13th) assessment of the six weeks training.

Angle-angle plots from the first session showed a low level of in-phase coupling between the pelvis and trunk, indicated by higher Areas of the convex hull. In the final (13th session) at the end of the sixth week the angle-angle plots showed tighter in-phase coupling with reduced Areas of the convex hull indicating a more synchronised rotation of the trunk and pelvis (Figure 3).

**Figure 4:**
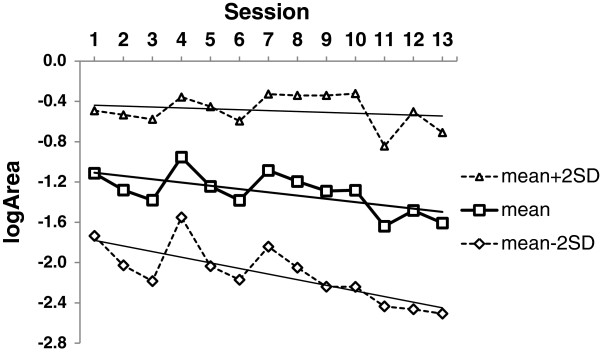
The reducing means of convex hull areas indicate increased coupling between the trunk and pelvis over the 13 game sessions.

Values of Area were not normally distributed, but a natural log transform corrected the distribution (logArea). The mean, maximum, minimum and standard deviation of logArea over all targets (trials) were calculated for each Session. Regression of mean logArea against Session showed that mean logArea reduced significantly over the training sessions (*F*_*1,11*_ *= 7.482, p = 0.019*), Figure 4. This, however, concealed more complex behaviour. The maximum logArea did not change over the training sessions, but the mean and minimum logArea reduced significantly as training progressed. As the maximum and minimum values are not unbiased measures of data spread values of Mean + 2 × STD and Mean - 2 × STD of logArea were used instead in regressions against Session. The difference in slopes seen with maximum and minimum values was again present (Figure 4: regressions: Mean + 2 × STD *F*_*1,11*_ *= 0.539,* p = 0.478, Mean - 2 × STD *F*_*1,11*_ *= 15.526,* p = 0.002).

**Figure 5:**
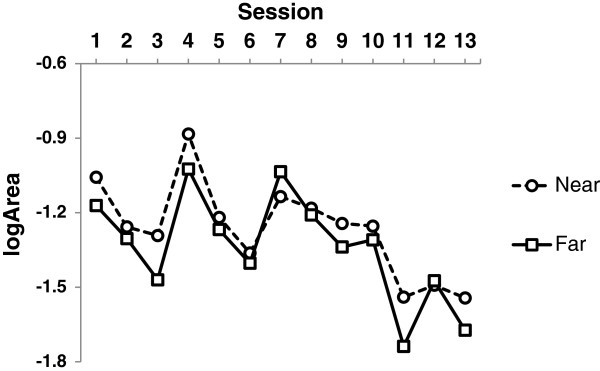
The convex hull areas indicate increased coupling (smaller logArea) between the trunk and pelvis when navigating to Far targets as compared to Near targets.

Due to their spatial distribution, reaching half of the balloon targets required less pelvic rotation (Near targets) than reaching the other half (Far targets), Figure 1. Separating the means of logArea for Near and Far targets for all the Sessions, a paired *t-test* showed that trunk to pelvis in-phase coupling is stronger for Far targets than for Near targets (Figure 5), *t*_*12*_ *= 3.259, p = 0.007*.

## Discussion

Over the period of six weeks while targeting movement training to the pelvis in a patient with cerebral palsy, a gradual development of increased coupling of the pelvis to the trunk was found. This strategy improved performance during gameplay which we have reported elsewhere [22] but improved selective movement control of pelvic rotation was not achieved. Previous findings indicate better movement control of the trunk in comparison to the pelvis in children with cerebral palsy during computer based game-play [25]. The tighter coupling of the pelvis to the trunk appears to be a compensatory mechanism which enables the child to improve control of the pelvis indirectly by locking it to the better controlled trunk.

Co-contraction, that is simultaneous activation of agonist and antagonist muscles, is a common motor control strategy used to improve stability and accuracy when performing an unpractised task [28]. Levels of co-contraction decrease over time with practice. Playing the training game twice a week for 30 minutes over 6 weeks appears to have resulted in the interlocking of the trunk and pelvis, representing the initial response to the new game task. Intensifying exposure to the game by means of increased frequency or duration of play, or both, may lead to reduced coupling between the trunk and pelvis as selective control improves.

Development of strategies compensating for impaired movement can be prevented by constraint induced movement therapy which physically inhibits the compensation thereby forcing use of the targeted movement [29]. Such a constraint preventing the use of trunk rotation while driving the GPO game with pelvic rotation can be provided by the game software and may be a means to facilitate use of the pelvis independent of the trunk. The GPO game can be set to either give a visual warning in case of trunk rotation or to be driven by the difference between pelvic and trunk rotation thereby preventing coupled motion. Initial tests however showed that typically developing children found it more difficult to play the game with such control schemes so they were not examined in the present study.

An increased level of coupling was found when using a large range of pelvic rotation needed to reach targets far from the midline. The child was able to move the pelvis with less in-phase coupling when small ranges were required (indicated by higher logArea) but pelvic movement was aided by in-phase coupled trunk rotation when reaching targets further away from the midline (confirmed by lower logArea). Over the duration of six weeks training the level of coupling increased but the difference of coupling over small and large ranges of pelvic rotations remained (Figure 5).

Functional measures of the patient showed mixed results when comparing performance before and after game training. On one hand the Segmental Assessment of Trunk Control scores increased (SATCo Static:2 → 4 Active:1 → 1 Reactive:NA → 2 [30]), the child’s parents commented on improved sitting posture and the swimming coach reported “improvements” in swimming. On the other hand an objective evaluation of the patient’s deviation from normality using the Gait Deviation Index [31] showed negligible changes in gait (75.6 → 75.0).

## Conclusions

Our case study of a child with cerebral palsy diplegia demonstrated that control was translated from the trunk to the pelvis through tighter coupling over six weeks training consisting of thirteen game sessions. Increased exposure to the game is expected to lead to reduction of coupling due to reduced co-contraction in muscles linking the trunk and pelvis as selective control improves. The Goblin Post Office game has means to facilitate selective control of the pelvis and this might reduce the time needed to achieve improved selective movements. Pelvic rotation is helped more by trunk rotation at the ends of the pelvic rotation range due to mechanical constraints and so training of selective pelvic control is likely to be more productive near to the neutral orientation.

## Competing interests

The first two authors have received financial support from Motek Medical (Amsterdam, The Netherlands) to attend user group meetings and scientific conferences. Their host university would receive a proportion of the income Motek Medical generates by sale of the Goblin Post Office game.

## Authors’ contribution

GJB conceived the study and drafted the manuscript. MBH participated in the design of the study and performed the statistical analysis. RJF carried out the data collection and analysis and helped to draft the manuscript. GJB, MBH and RJF developed the Goblin Post Office game with contribution from GH and PBB on the conceptual and practical aspects of targeted training. All authors read and approved the final manuscript.
